# Linking Ethical Leadership to Followers’ Knowledge Sharing: Mediating Role of Psychological Ownership and Moderating Role of Professional Commitment

**DOI:** 10.3389/fpsyg.2022.841590

**Published:** 2022-02-10

**Authors:** Imran Saeed, Jawad Khan, Muhammad Zada, Shagufta Zada, Alejandro Vega-Muñoz, Nicolás Contreras-Barraza

**Affiliations:** ^1^Institute of Business and Management Sciences, The University of Agriculture, Peshawar, Pakistan; ^2^Department of Business Administration, Iqra National University, Peshawar, Pakistan; ^3^Business School, Henan University, Kaifeng, China; ^4^College of Education, Henan University, Kaifeng, China; ^5^Department of Business Administration, ILMA University, Karachi, Pakistan; ^6^Public Policy Observatory, Universidad Autónoma de Chile, Santiago, Chile; ^7^Facultad de Economía y Negocios, Universidad Andres Bello, Viña del Mar, Chile

**Keywords:** ethical leadership, knowledge sharing, professional commitment, psychological ownership, social learning theory

## Abstract

This study examined (1) the influence of ethical leadership on knowledge sharing, (2) the mediating role of psychological ownership, and (3) the moderating effect of professional commitment between ethical leadership (EL) and knowledge sharing (KS). Data were collected from 307 public listed Pakistani companies’ employees. Statistical analyses were performed by using SPSS Version 25 and AMOS version 22. The findings indicate a positive relationship between EL and KS behavior. Additionally, the impact of EL on KS was partially mediated by psychological ownership. Furthermore, professional commitment buffers the link between EL and KS. This study contributes to the body of knowledge in the field of leadership by confirming the role of ethics. The results show that ethical leaders develop employee attitudes (i.e., psychological ownership and professional commitment) that contribute to employee KS behavior. Ethical leaders create and encourage a learning culture to enhance organizational performance. This study adds to the little data on the positive impact of EL on listed company’s employees and addresses the gaps in previous studies on the role of EL in changing environments. In addition, professional commitment as a moderator has not been previously investigated with ethical leadership antecedents.

## Introduction

Leaders are credited with helping their organizations in getting an edge in the marketplace by managing their finances and teaching ethical values to the employees (Koay and Lim, 2021; Yasin, 2021). Over the last decade, ethics in the workplace have received increased attention from researchers, who have long acknowledged the relevance of ethics in employee’s character development (Van Gils et al., 2015). Behavioral ethics and ethical leadership (EL) have received a great deal of attention in the wake of the Enron and National Irish Bank scandals in 2001, owing to the long-term repercussions they may have on an organization (Ali et al., 2018). Leaders play a critical role in enhancing staff morale and increasing productivity (Fatima et al., 2017).

*EL is defined as “the demonstration of normatively appropriate conduct through personal actions and interpersonal relationships, and the promotion of such conduct to followers through two-way communication, reinforcement, and decision-making”* Brown et al. (2005).

Social learning theory is one of the most frequently referenced theories to study the link between EL and followers conduct in the social learning workplace (Keen et al., 2005). We advocate an additional mechanism to better understand the complicated link between EL and employee behavior. Researchers have examined a wide range of factors that results due to ethical leadership. One of the main factors that helps employees to spread, learn and taught the basic skills, knowledge and abilities (i.e., knowledge sharing). EL effects on employee’s knowledge sharing behavior was studied from a social perspective (Ganguly et al., 2019), personal characteristics (Kim and Shim, 2018), and cultural influences (Liu et al., 2018). Ethical leadership has significant relationship with knowledge sharing (Bhatti et al., 2021). Most studies have demonstrated that leadership significantly motivates individuals to share their knowledge with colleagues, regardless of the organizational setting (Ali et al., 2021). A recent study also found that ethical leadership reduce employee knowledge hiding behavior and increase employee wellbeing and knowledge sharing behavior (Agarwal et al., 2022). Organizational effectiveness and success are directly linked to EL and knowledge sharing (KS) connection and also linked to leaders’ ability to advise, organize, motivate, and empower their followers (Le and Lei, 2018). Most studies on leadership focus on how leaders influence their subordinates, rather than how their subordinates get advantage from their leadership guidance and supervision (Su et al., 2021). There is a dire need to find that how knowledge sharing behavior can be enhance in the presence of individual characteristics (Luo et al., 2021). Thus, our study suggests that subordinate characteristics may act as moderators in the EL development process to share information. Professional commitment (PC) was chosen as a moderator because it has emerged as a critical driver of employees’ attitudes and organizational success (Ghani et al., 2020). To complete the global market, organizations seek and encourage continuous innovation in operations and promote an agile workforce, along with their professional commitment to their job roles, to meet organizational objectives in an unpredictably changing environment. Organizations’ extra-role performance depends on employees’ loyalty to their profession and sense of responsibility toward organizational problems and challenges (Guerrero et al., 2017; Ghani et al., 2020). A company’s competitive advantage can no longer be gained from employees who do not fulfill their assigned tasks. Rather, employees’ dedication, and devotion to their work responsibilities are important factors to consider (Gerpott et al., 2019). In today’s workplace, professional commitment has emerged as one of the most essential factors in encouraging employees to be proactive and has evolved as a main source of motivation to share knowledge with others (Chang et al., 2019).

*“Professional commitment is defined as loyalty, the desire to stay in a profession, and a sense of responsibility toward the profession’s particular problems and challenges*” (Mitchell et al., 2019).

Employees who hold high ethical standards from their ethical leader will play an active role in shaping the organization’s goals and assisting their fellow workers by sharing and assisting in the transmission of relevant information related to the organization’s objectives (Bavik et al., 2018). This study also investigated how EL effects and helps employees in developing feelings of ownership to gain control over a target through guidance and knowledge. The likelihood of employees feeling a strong sense of psychological ownership (PO) in their jobs increases when they work under morally good leadership (Mishra and Malhotra, 2021). Furthermore, it is critical to emphasis the value of psychological ownership as a driving force for employee voice and knowledge exchange (Sun et al., 2019).

Theoretical and practical applications are relevant to this study. From a theoretical standpoint, our study makes a significant contribution to the corpus of knowledge on EL and knowledge sharing. First, we examined whether a company’s KS culture is influenced by its leadership’s ethical standards. Despite the fact that KS is increasingly being acknowledged as a psychological behavior in an organizational environment. Research on the psychological aspects of employees’ capacity to share knowledge has not been conducted under changing circumstances (e.g., COVID 19) extensively. This is the first study to examine how EL influences the development of workers’ ability to share knowledge during the changing environment. Second, we used social learning theory to show how strong employee professional commitment when combined with EL, leads to a willingness to share knowledge among colleagues. Third, by recognizing KS as a psychologically and interpersonally significant phenomenon, this study investigated the significance of EL as a predictor of information exchange in organizations. For the knowledge management literature, this is a fresh approach to understanding employees’ attitudes. From a practical perspective, our findings demonstrate that leaders play a critical role in promoting an information-sharing culture. Organizations that want to maximize the value of their intangible assets by increasing the use and retention of workers’ expertise and information *via* KS would benefit the organization in long run. EL is also being studied in a new way, as earlier research has focused on the qualities and personality traits of leaders without taking into account the effect of follower traits that learned from the leader (Sharif and Scandura, 2014; Karim and Nadeem, 2019; Mostafa et al., 2021). This study has the importance in Yarn production sector in Pakistan. Yarn production sector is one of the most growing sectors of developing country like Pakistan. This study will open new avenue in Yarn production sector and will add more knowledge to the body of literature in the area of ethical leadership and knowledge sharing behavior.

## Theory and Hypothesis Development

### Social Learning Theory

**Figure 1:**
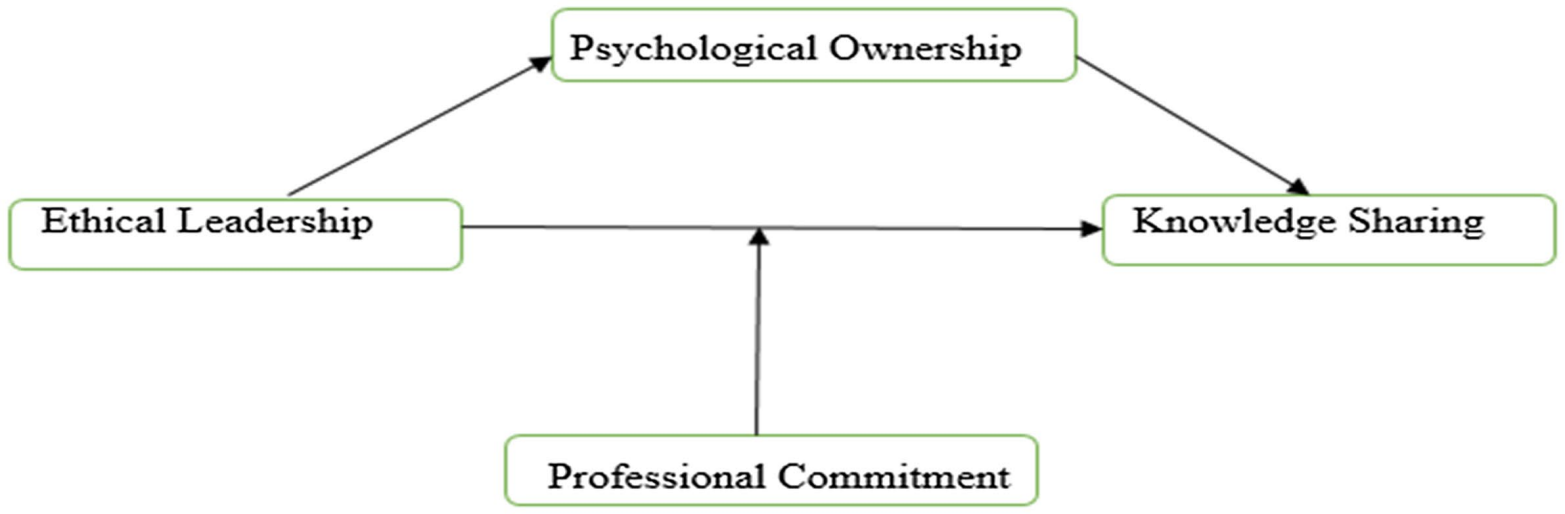
Conceptual model.

Grounding on social learning theory (Bandura and Hall, 2018), ethical leaders have an influence on their employees *via* observational learning, in which employees learn indirectly by seeing the activities and repercussions of ethical leaders’ actions (Bandura, 1979; Grusec, 1994; Brown et al., 2005). Similarly, we contend that ethical leaders have an effect on the behavior of their subordinates’ psychological wellbeing *via* the social learning process. With regard to social learning, “the psychological states of the receivers decide which external events to be considered, how they are interpreted and if they leave untouched what will be the consequences” (Bandura, 1979). A person’s ability to pay attention, analyze, and react to ethical leaders’ actions may be hindered by psychological conditions that produce cognitive dysfunction (Brown et al., 2005). More recent literature focuses on leadership and psychological ownership relationship mechanism and revealed that leadership is the main cause root of psychological ownership (Guarana and Avolio, 2022). Prior social learning theory research has paid little attention to followers’ psychological states (Bavik et al., 2018), particularly within the EL framework. We looked at psychological ownership in order to better understand the role of psychological states that emerged during the social learning process (Wright and Cropanzano, 1998) and argued that it is likely to enhance the connection between EL and KS behavior. In other words, the study’s second goal is to determine whether employee professional commitment acts as a buffer between EL and knowledge-sharing in the social learning process (see Figure 1).

### Ethical Leadership and Knowledge Sharing

In an organization, KS does not cause automatically; it requires a certain set of circumstances and ongoing assistance. Information sharing is likely to be significantly influenced by leaders’ actions (Ali et al., 2018). Leader support is critical for fostering a culture of knowledge sharing among company workers (Tang et al., 2015). EL is considered an effective leadership style (Bedi and Wordley, 2019), and is linked to KS behaviors (Su et al., 2021). As part of knowledge management, the goal is to establish an environment that encourages individuals to share information and build a shared understanding of the company’s mission. EL significantly influences individual participation and attitudes toward information sharing (Koay and Lim, 2021).

*KS refers to “acts of making knowledge available to others within the organization”* (Ipe, 2003).

Leadership has been found to have a substantial influence on the interpersonal process of knowledge exchange within organizations (Xue et al., 2011; Rahman et al., 2019). The psychological impact of KS leads to the belief that ethical leader behavior is an important predictor. Fairness, transparency, and trustworthiness are essential components of effective EL that needs for learning culture (Bhatti et al., 2021). Ethical leaders can motivate followers to share their expertise (Wu and Lee, 2017; Men et al., 2020). As a first step, ethical leaders help to reduce the physical barriers that prevent employees from sharing resources by incorporating processes and controls that encourage employees psychologically (e.g., code of ethics, standards for making ethical decisions, open two-way communication, and a fair incentive system) are all important components of an ethical workplace. Second, when ethical leaders exemplify the values and standards of their respective organizations by behaving in line with these values and standards, they demonstrate their values and norms (such as trustworthiness, kindness, and concern for the needs of others; Banks et al., 2021). Based on these considerations, EL encourages employee KS by eliminating organizational obstacles, cultivating trustworthy workplace connections, and fostering workers’ expectations of fair compensation for their contributions and resource sharing (Castellani et al., 2021). The knowledge management literature has demonstrated the importance of leadership in establishing rules and directions that encourage KS (Lin et al., 2020). Several studies have indicated that ethical leaders motivate their followers to participate in pro-social activities, such as sharing knowledge and assisting others to gain knowledge (Gerpott et al., 2020; He et al., 2021), while reducing deviance (Evans et al., 2021; Khan et al., 2022).

*Hypothesis 1*: EL has significant and positive effect on KS.

### The Mediating Role of Psychological Ownership

*“Employees will put forth better efforts to care for, nurture and maintain things which they consider to be their own. When individuals have a sense of ownership, they feel connected with each other in achieving several tangible and intangible targets* (Dittmar, 1992)*.”*

Employees’ psychological ownership and EL are intertwined in the same way through social norms (Kim and Beehr, 2017). By highlighting key principles and norms that are connected to psychological ownership, ethical leaders are likely to impact the behavioral norms of the organizations (Ali et al., 2021). A growing number of moral leaders are particularly concerned about protecting the rights of their workers and obligations when it comes to the first value for equality. There is a strong feeling of commitment to job duties, as well as a strong sense of right to influence the outcomes of work when one takes an ownership perspective on things (Adil and Kamal, 2018; Xiao and Cooke, 2019). Employee ownership was eliminated if the rights were violated. However, because they expressly seek justice, ethical leaders are less likely to violate the supposed rights, equality, and compassion (Brown et al., 2005; Gul et al., 2021). In other words, if an employee has a significant stake in a project, the ethical leader is inclined to remain on the board. However, unethical leaders are less inclined to do this. Ethics-based leadership is more likely to demonstrate and foster responsibility among followers, ensuring that employees’ rights are protected. Brown et al. (2005) stated that ethical leaders penalize workers who break ethical norms and hold employees responsible for their work performance. According to the social learning theory, ethical leaders produce positive and productive results through social learning and two-way communication (Bandura, 1979). According to Bandura and Hall (2018), direct and indirect interactions can help employees develop a sense of accountability, such as seeing ethical leaders holding individuals responsible for their accomplishments as well as the method by which those successes were obtained. The norms for social conduct in the organization were established based on observations and interactions between employees (Cheng et al., 2021). In addition, employees are held responsible for their own actions (Podsakoff et al., 2003). Employees who work with morally conscientious bosses are likely to have psychological control over their actions. Ethical leaders create a sense of belonging in the workplace and instill a sense of psychological ownership in their subordinates. Ethical leaders should focus on their workers by listening to them and giving them a voice in the workplace (Brown et al., 2005). Employees who are given the opportunity to voice their opinions and have a voice in the design of their workplace are more likely to feel like part of the team as a whole and are more willing to share their knowledge and experience, as suggested by the job characteristics model and supported by Pittino et al., (2018) and Spreitzer (1995). As a result of being neglected, workers might become emotionally disengaged and feel that they do not belong to the organization, and they are more inclined to keep their knowledge hidden from the rest of the organization (Arain et al., 2020). When ethical leaders place their followers’ interests first and listen to their concerns, they experience a feeling of belonging in the workplace, which is a key component of psychological ownership that encourages them to share their knowledge (Men et al., 2020). The link between psychological ownership and KS entails the conversion of human perceptions and sentiments into an organizational stance. As previously stated, the formation of stewardship culture inside a company may facilitate KS (Pittino et al., 2018). Psychological ownership may motivate individuals to share knowledge, encourage others to learn, work together to solve issues, accept and propagate organizational values and ideas, and accept and disseminate organizational values and ideas (Gupta et al., 2020; Singh et al., 2021). A similar study by Hameed et al. (2019) argued those employees who have high level of psychological ownership shows altruistic spirit and enhance KS and it is considered one of the crucial antecedents of knowledge sharing.

*Hypothesis 2*: Psychological ownership positively and significantly mediates the link between EL on KS.

### The Moderating Role of Professional Commitment

*“Professional commitment is defined as a psychological attachment to one’s profession* (Aranya et al., 1981; Wallace, 1995).*”*

Previous studies have identified a substantial correlation between a person’s commitment to their profession and willingness to share their expertise (Chen et al., 2020; Zada et al., 2021). Additionally, KS may be nurtured and developed if employees have developed favorable attitudes and gestures toward an organization, which requires a lot of internal desire (Khan and Ali, 2019; Tahir et al., 2019). Ethical leaders play an important role in shaping and assisting the professional lives of their workers so that they can participate in KS behavior (Kuenzi et al., 2019). According to Bavik et al. (2018), despite the fact that EL conduct affects workers’ attitudes toward KS, there have been few studies on the psychological effects of such activities.

Previous research has shown that leaders are substantially connected with KS behaviors and have pivotal responsibilities in the success of the business by psychologically influencing workers to spread information (Wu and Lee, 2017). Individuals who are supported by ethical leaders are more likely to promote and share their expertise with others, therefore, broadening the breadth of KS, according to our research. Developing a knowledge-sharing culture requires a strong commitment from workers (Lei et al., 2019) to share and receive information (Le and Lei, 2018). Knowledge exchange and professional commitment are typically assumed to be linked. According to Bavik et al. (2018), greater dedication encourages employees to share new ideas. According to Han et al. (2010), are more willing to share their knowledge and experience with their colleagues. Based on the study by Alrawi et al. (2016), KS is more effective when several workers are involved. Lin (2007) also identified a correlation between professional dedication and tacit KS was also identified by Lin (2007), which is in line with earlier studies.

*Hypothesis 3*: Professional commitment positively and significantly strengthens the relationship between EL and KS, such that the relationship is stronger when professional commitment is high.

## Methodology

### Sample and Procedure

To meet the study aims, a survey technique was used to obtain quantitative data from the participants. The intended audience consisted of individuals who worked in publicly listed yarn production sector companies in Pakistan. The reason behind to select this company as one of our team members working in administrative post and there was easy to collect data from these organizations. They must, however, satisfy certain requirements in order to be considered for participation in our survey. First, they must have at least one direct supervisor to whom they must report to function properly. Second, each supervisor must provide feedback of four subordinates. This is due to the fact that our target populations were asked to assess ethical support received from their line managers as part of their job roles. It is difficult to succeed without reporting to a direct supervisor or a leader. Preliminary testing of the survey questions on three academic experts was conducted before they were sent to the real target population to confirm that all of the scales under investigation had positive face validity. Two academic experts were from specific field of management sciences and one from statistics field in order to verify the scale. All constructions have Cronbach’s *α* values higher than 0.7, which indicates that all scales are reliable according to preliminary data of 28 respondents collected for piloting testing (Iacobucci and Duhachek, 2003). After clarifications were made regarding the questionnaire, every public listed company in Pakistan that was approached through personal contact received a link to the online survey. We asked the person in charge of the distribution to pass the link with the employees. However, we clarified to the representative that to be considered for our target responders, workers had to meet the criteria listed above. The data collection period was split into two periods separated by 15 days in accordance with the suggestions of Podsakoff et al. (2003) to help in the reduction of common method bias, which was followed. First, we gathered information from independent and mediating variables and demographic characteristics, in the second phase, we gathered information from dependent and moderating variables. In the first phase, 420 employees were contacted and 364 questionnaires were received (86.66%). In the second phase, we received 316 questionnaires out of 364 questionnaires (86.81%). After thoroughly studying the questionnaires, we excluded nine questionnaires due to missing data. In total, 307 completed data were collected. The respondents included 264 men (85.99%) and 43 women (14%) females. In terms of age group, 37 (12.05%) were between 25 and 29 years old, 137 (44.62%) were 30 and 35, 98 (31.92%) were 36 and 40, and 35 (11.40%) were 40 and above. Education statistics showed that 12 (3.90%) had completed HSSC, 72 (23.45%) had a bachelor’s degree, 192 (62.54%) had a master’s degree, 27 (8.79%) had an MS/Phil qualification, and 4 (1.30%) had a doctorate. The full demographic profile of the respondents is presented in Table 1.

**Table 1:** Sample characteristics.

Demographic variables	Frequency	Percentage
**Gender**		
Male	264	85.99
Female	43	14.00
**Age**		
25–29	37	12.05
30–35	137	44.62
36–40	98	31.92
Above 40	35	11.40
**Experience**		
1–5	176	57.32
6–10	62	20.19
11–15	42	13.68
Above 16	27	8.79
**Qualification**		
HSSC	12	3.90
Bachelor	72	23.45
Master	192	62.54
MS/Phil	27	8.79
PhD	4	1.30

### Measures

#### Ethical Leadership

We used a 10-item scale created by Brown et al. (2005) to assess EL. Examples of such items are: “my supervisor makes fair and balanced decisions,” “when making decisions, asks “what is the right thing to do?,” and “disciplines employees who violate ethical standards” Internal reliability (*a* = 0.94) was found to be appropriate for this instrument. All items were measured in 5-likert scale.

#### Psychological Ownership

Avey et al. (2008), 12-item scale with 5-likert scale was used to assess psychological ownership. Sample items are “I feel this organization’s success is my success” “I am totally comfortable being in this organization,” and “I am confident I can make a positive difference in this organization.” Internal reliability (*a* = 0.90) for the psychological ownership measure was satisfactory.

#### Professional Commitment

Four scale items with 5-likert scale from a prior study on healthcare professional commitment were included in the member questionnaire to gage members’ level of professional commitment (Lachman and Aranya, 1986; Chang and Choi, 2007; Teng et al., 2009). Sample items are “the extent to which they felt strong ties with their professional group”; “felt closely connected to their professional group”; “felt happy to be a member of their profession.” The Cronbach *α* for professional commitment was (*a* = 0.95).

#### Knowledge Sharing

The employees’ KS behavior was assessed using Connelly et al. (2012) five-item KS with 5-likert scale. Sample items are “This coworker looks into my requests to make sure his/her answers were accurate.” “This coworker explains everything very thoroughly.”

## Pretesting

### Common Method Variance

In survey-based investigations, common method variance (CMV) should be investigated, particularly when data for independent and dependent variables are obtained simultaneously using the same technique at the same time. As advised by MacKenzie and Podsakoff (2012), we used procedural remedies that they suggested to overcome CMV, such as incorporating a well-written cover letter and ensuring that the respondents’ privacy was protected. This study also included several statistical tests to measure the severity of CMV. Upon closer examination of Table 2, it becomes clear that there is no significant link between the two variables above 0.9, suggesting that the CMV is not a reason for concern. Second, we used Kock (2015) full collinearity test, which was modified significantly. As shown in Table 3, all variance inflation factor values in this investigation were less than 3.3, indicating that CMV risk was not found in this study.

**Table 2:** Results of the confirmatory factor analysis (*N* = 307).

Model	χ2/df	RMR	GFI	CFI	RMSEA
Baseline model (four-factor model)^o^	2.23	0.03	0.88	0.90	0.02
4-factor model^a^	4.57	0.07	0.93	0.91	0.07
3-factor model^b^	2.57	0.06	0.91	0.90	0.08
2-factor model^c^	1.62	0.05	0.94	0.91	0.07
1-factor model^d^	5.57	1.04	0.47	0.37	0.18

a Combining EL&KS.

b Combining EL, KS&PC.

c Combining PC, PO&KS.

d PC, PO &EL.

o Combining all items.

**Table 3:** Factor loadings.

Constructs	Items	Factor loadings	√AVE	CR	AVE
	EL1	0.86			
	EL2	0.85			
	EL3	0.89			
Ethical leadership	EL4	0.76	0.72	0.94	0.53
	EL5	0.73			
	EL6	0.81			
	EL7	0.72			
	EL8	0.75			
	EL9	0.82			
	EL10	0.78			
	KS1	0.84			
Knowledge sharing behavior	KS2	0.78	0.80	0.90	0.64
	KS3	0.81			
	KS4	0.77			
	KS5	0.82			
	PO1	0.84			
	PO2	0.92			
	PO3	0.91			
	PO4	0.78			
	PO5	0.83			
Psychological ownership	PO6	0.71	0.81	0.95	0.66
	PO7	0.91			
	PO8	0.81			
	PO9	0.82			
	PO10	0.66			
	PO11	0.88			
	PO12	0.67			
	PC1	0.85			
Professional commitment	PC2	0.82	0.80	0.88	0.64
	PC3	0.79			
	PC4	0.76			

### Measurement Model

In the assessment criteria presented by Hair et al. (2017) internal consistency, reliability, convergent validity, and discriminant validity are all reviewed as part of the evaluation process. As shown in Table 4, all constructs had Cronbach’s *α* values more than 0.7, and the composite reliability values in Table 3 were greater than 0.7, showing that variables had excellent internal consistency. Additionally, since the factor loadings were larger than 0.7 and the average variance extracted (AVE) values of all reflective components were greater than 0.5, convergent validity was established. To establish discriminant validity, the Fornell–Larcker criteria and the Heterotrait–Monotrait ratio (HTMT) criterion were utilized. We found that AVE’s square root was greater than the correlation values in the rows and columns to fulfill the Fornell–Larcker criterion (Table 3). The HTMT number should not exceed 0.85 in terms of the HTMT criterion (Henseler et al., 2015). Table 4 shows that all of the HTMT values in this study were less than 0.85, indicating that discriminant validity was not a major problem.

**Table 4:** Correlations, mean, standard deviation and reliability.

Variables	Mean	SD	HTMT			VIF	1	2	3	4	5	6	7	8
1. Gender	1.3616	0.48124												
2. Age	2.4463	0.82815					−0.078							
3. Education	2.8664	0.58165					0.021	0.063						
4. Experience	1.9902	0.71165					−0.066	0.041	0.052					
5. EL	3.8446	0.72418	–			1.335	0.030	0.036	0.067	0.022	**(0.81)**			
6. PO	3.9742	0.70059	0.65	–		1.453	0.009	0.088	0.086	−0.043	0.572^**^	**(0.78)**		
7. PC	4.0008	0.80275	0.56	0.61	–	1.527	0.014	0.095	0.077	−0.066	0.493^**^	0.556^**^	**(0.77)**	
8. KS	3.8573	0.67975	0.47	0.57	0.61	1.627	0.008	0.033	0.016	−0.034	0.593^**^	0.674^**^	0.668^**^	**(0.76)**

** Correlation is significant at the 0.01 level (2-tailed). Cronbach's alpha highlighted as bold, all values >0.7.

### Confirmatory Analysis

A confirmatory factor analysis (CFA) was conducted to assess the factor structure of the EL, KS, psychological ownership, and professional commitment scales. Different confirmatory factor analyses were performed through AMOS 22. The resulting 4 factor model demonstrated excellent fit, *χ*2/df = 2.23, RMR = 0.03, GFI = 0.88, CFI = 0.90, and RMSEA = 0.02. See Table 2 for the series of CFA and see Table 3 for the factor loadings.

### Descriptive Statistics

Table 3 displays the means, standard deviations, correlations, and scale reliability of the data. The research variables’ correlations were in the predicted directions, and all of the study variables had an acceptable degree of internal consistency, as shown in Table 1. Employees’ KS behavior was positively related to EL (*r* = 0.668, *p* < 0.01). Furthermore, EL was positively related to both PO and PC (*r* = 0.572, *p* < 0.01; *r* = 0.556, *p* < 0.01). The Variance Inflation Factor was used to assess for multicollinearity in this study (VIF). The VIF values in Table 4 varied from 1.335 to 1.627, which is less than 2 and deemed to be within acceptable bounds (Hair et al., 1998).

### Control Variables

Several variables were kept under control. Individual demographics (such as age, gender, and educational level) have been found to impact employees’ knowledge behaviors in the past (Scholz and Schöner, 1999; Spector and Brannick, 2011; Connelly et al., 2012). As a result, these factors were brought under control in this study. We used four answer choices to control for individual employees’ educational level (1 = HSSC; 2 = bachelor’s; 3 = master’s; 4 = MS/Phil; 5 = PhD). The gender of the participants was dummy coded, with females being tagged as 1 and males being coded as 2. In addition, the size of the teams in our research was kept under control. According to previous studies, a bigger team size reduces a leader’s capacity to influence individual employee behavior as well as KS within workgroups (Zhao et al., 2016).

## Hypothesis Testing

### Direct Relationship and Mediation Analysis

Table 5 shows the impact of EL on employees’ KS. In Table 5, the *R*^2^ value of 0.35 revealed that EL explained 35% of the variation in KS behavior with *F*(1, 305) =165.70, *p* < 0.001. The findings revealed that EL was positively related to KS behavior (*β* = 0.55, *p* < 0.001). In Table 6, Hayes’ PROCESS (5000bootstrappingwasspecified) was used to test Hypotheses 2, EL was found to have a positive indirect (PO) relationship with KS *via* psychological ownership (*B* = 0.42, Lower limit = 0.3432, and Upper limit = 0.5102), showing that Hypothesis 2 was also accepted.

**Table 5:** Direct path.

	*t*	*R* ^**2**^	SE	*β*
EL→KS	12.8	0.35	0.54	0.557^***^

*** Correlation is significant at the 0.001 level (2-tailed).

**Table 6:** Mediation path.

	BootLLCI	BootULCI	Boot SE	*β*	Decision
Mediation path	0.3432	0.5102	0.0428	0.4259	Partial mediation

### Moderation Analysis

**Figure 2:**
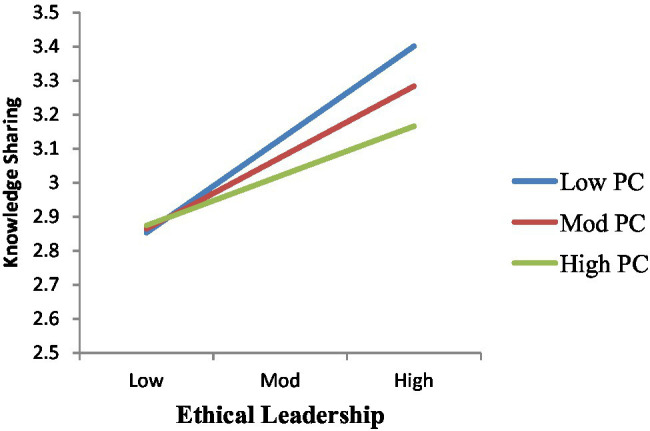
EL*PC=KS.

The moderating effect of PC in the relationship between EL and KS was investigated using Hayes (2017) (model 1). As a consequence of the findings (Table 7), it was found that the interaction term had a statistically significant effect on employees’ KS behavior (*β* = −0.1046^*^), indicating that PC moderated the positive relationship between EL and employees’ KS behavior. In Figure 2, the interactions between EL and KS and are shown at ± standard deviation from the mean of PO. A simple slope test was used to determine the strength of the positive associations between EL and employees’ KS behaviors at high and low levels of PC. The simple slope test showed a significant positive relationship (*β* = 0.1771, *p* = 0.0007) for employees with high PO, Thus, Hypothesis 3 is verified.

**Table 7:** Results of the moderated path analysis.

Ethical leadership*psychological ownership = knowledge sharing							
	*Β*	SE	*t*	*p*	95% CI		
					LL	UL	
EL	0.6794	0.1575	4.3125	0.0000	0.3694	0.9894	
PC	0.8923	0.1441	6.1939	0.0000	0.6088	1.1758	
EL*PC	−0.1046	0.0394	−2.6549	0.0084	−0.1821	−0.0271	
HP (−1 SD)	0.3450	0.0458	7.5291	0.0000	0.2548	0.4352	
HP (+1 SD)	0.1771	0.0516	3.4350	0.0007	0.0756	0.2786	
*R* ^2^							0.64
Δ*R*^2^							0.0082

## Discussion

First, we found that EL was linked to employees’ willingness to share their knowledge, which is consistent with social learning theory (Bandura, 1979; Bandura and Hall, 2018). We also examined the moderating influence of professional commitment and how EL may help employees in their professional lives by encouraging them to share knowledge with coworkers. Furthermore, this study investigated the significance of psychological ownership in mediating the relationship between EL and KS behavior. Our findings have substantial consequences for both theory and practice in the corporate environment.

### Theoretical Contributions

First, this study examines the effect of EL on employees’ knowledge-sharing behavior using a psychological perspective. In spite of previous research indicating that EL has a significant impact on employees’ risk-taking and pro-social behavior, such as employee voice, creativity and organizational citizenship, there is surprisingly little research on the impact of EL on employees’ KS (Lei et al., 2019; Men et al., 2020). Sharing one’s knowledge is primarily a risk-taking and pro-social behavior with a psychological component, as it implies that one’s willingness to share knowledge with others will be reduced if one’s ownership of one’s expertise is lost (Spender and Grant, 1996). Our findings show that EL is important for encouraging employees to share their knowledge. This finding adds to the growing body of evidence that information sharing has psychological significance. Future studies could examine additional potential antecedents of information sharing at work from a psychological standpoint. Second, most previous studies on EL have relied on theoretical perspectives, such as social exchange theory (Garba et al., 2018; Wang et al., 2019; Eva et al., 2020) and social identity theory (De Roeck and Farooq, 2018; Yang and Wei, 2018; Gerpott et al., 2019). We investigated the impact of EL on KS among workers using the social learning theory to explain the influence of EL on employee behavior. Our research sheds light on the mediating mechanisms of psychological ownership as well as the moderating variable of professional commitment by demonstrating that employees engage in pro-social and psychological behavior under the guidance and supervision of EL to share knowledge. According to Garba et al. (2018), as a manager and as a role model, an ethical leader actively enhances the external events of the organization while also transforming the self-Concept of their employees. This finding supports the theoretical concepts. When it comes to psychologically strong employees and management traits, our study provides a more complete picture of how these two aspects interact to influence employee behavior in a more comprehensive manner. Using this approach, we can provide empirical evidence to support our theoretical understanding of EL by demonstrating that a leader’s twin functions as a psychological person and psychological manager are crucial in encouraging colleagues to act in a professional manner (Adil and Kamal, 2018; Xiao and Cooke, 2019). Third, as a result of our research, we found that employee professional dedication and KS among colleagues are both critical variables in encouraging information sharing among colleagues. According to the results, EL is a value-based leadership style generally acknowledged in the development of workers’ professional skills and abilities (Den Hartog, 2015). In the case of an ethical leader who is a real ethical role model, the values of that leader are recognized and reflected by their followers’ behaviors.

Our findings also demonstrate that professional commitment is associated with an explanatory power that is comparable to psychological ownership in the EL- employee KS relationship. As Ryan and Deci (2002) argue, external aims are not necessarily inferior to internal driving factors when it comes to motivating people’s behavior (Eva et al., 2020). Our findings corroborate the efficacy of the twin processes (psychological ownership and professional commitment) predicted by the EL paradigm is significant affects followers’ attitudes and conduct to a great degree. Our theoretical model meaningfully connects the literature on leadership, knowledge management, psychological ownership, and professional commitment, and more research is needed to fully understand the impact of EL on the psychological wellbeing of others around them, as this study demonstrates.

### Practical Implications

Our research provides insight into how organizations might encourage their employees to share their knowledge in ways that are beneficial to the company as a whole, rather than just to themselves. First, according to our findings, ethical leaders are actively involved in stimulating information sharing among their staff. Employees who desire to boost KS at work may want to be mentally robust and to participate in professional development programs that encourage ethical conduct. Second, organizations could encourage EL by offering training programs for leaders, stressing the relevance of psychological concepts, and presenting examples of ethical behavior that leaders should demonstrate in their everyday behavior and management practices. Third, when it comes to encouraging information sharing within an organization, it is critical that employees understand the significance of both internal and external regulations. Consequently, organizations must regularly implement mental health programs to prevent interruptions in the flow of information among their employees.

## Limitations and Future Avenues

There are several possible drawbacks to this research as well. First, all factors, such as EL, psychological ownership, professional commitment, and KS, were derived from the same source. Because the data were acquired from the same source, a common technique bias may have emerged (Podsakoff et al., 2003). Experimental designs that enhance causal inference should be used in future studies to address this issue. Second, a theoretical model was developed and evaluated at both individual and team levels. Several control variables have been included at both the individual and team levels. Individual and team-level parameters, such as age and gender were considered in our investigation. As Serenko and Bontis (2016) argue, organizational factors like organizational culture, which may affect people’s motivation to share information, may also have an impact on individuals’ willingness to share knowledge. Corporate culture control should be the primary focus of future research as well as identifying whether such a theoretical model is validated at the organizational level of examination. Third, in accordance with social learning theory, we investigated a mechanism that connects EL with KS in organizations. However, other possible mechanisms cannot be ruled out. As KS progresses, it is necessary to investigate various possible models using a variety of theoretical approaches. For example, may lead to a greater desire to share knowledge (Huo et al., 2016). Future studies should incorporate this behavior. In addition, there may be other possible moderators in the link between EL and KS, such as self-Monitoring, political skills as well as conscientiousness, societal norms, and individual variations based on morality (Bavik et al., 2018). Self-Monitoring, political skills, and conscientiousness have been shown to increase the likelihood that employees will share their knowledge with their coworkers because they see it as a professional obligation. EL and KS should be examined in light of these moderating effects in future research (Bavik et al., 2018).

## Final Thoughts

Most organizations find it challenging to motivate employees to share their knowledge and abilities with colleagues in a productive and effective manner. In the disciplines of organizational behavior and knowledge management, the results of this research may be used to better understand the links between EL, organizational performance, trust in leaders, and certain aspects of knowledge behavior in the workplace. The findings of this study support the hypothesis that EL is crucial in improving employees’ psychological wellbeing and developing employees’ loyalties to their jobs and organizations, allowing them to share knowledge with their colleagues. KS behavior can be strongly influenced by EL. The fact that a leader treats employee with dignity, honesty, fairness, and integrity, enables them to participate in decision-making; and encourages them to do so and the fact that the leader encourages normative and ethical behavior among his or her workers *via* two-way communication, may contribute to followers’ good evaluations of their leader’s personality. This research examines and determined that EL is an effective approach to encourage KS among employees by using social learning theory. Our findings offer preliminary empirical evidence in favor of the theoretical model of EL as a foundation for learning organizations. To create an effective learning environment and encourage resource sharing among employees, these results emphasis the need for ethical leaders who not only support their followers psychologically but also act as a mentor to help them in developing their professional commitment.

## Data Availability Statement

The raw data supporting the conclusions of this article will be made available by the authors, without undue reservation.

## Ethics Statement

Ethical review and approval was not required for the study on human participants in accordance with the local legislation and institutional requirements. The patients/participants provided their written informed consent to participate in this study.

## Author Contributions

JK, IS, and MZ contributed to the conception and design of the study. SZ organized the database. JK performed the statistical analysis. JK, IS, MZ, and SZ wrote the first draft of the manuscript. AV-M and NC-B wrote sections of the manuscript. All authors contributed to manuscript revision, read, and approved the submitted version.

## Funding

Funding for the publication fees of this article have been provided by Universidad Autónoma de Chile and Universidad Andrés Bello (NC-B and AV-M).

## Conflict of Interest

The authors declare that the research was conducted in the absence of any commercial or financial relationships that could be construed as a potential conflict of interest.

## Publisher’s Note

All claims expressed in this article are solely those of the authors and do not necessarily represent those of their affiliated organizations, or those of the publisher, the editors and the reviewers. Any product that may be evaluated in this article, or claim that may be made by its manufacturer, is not guaranteed or endorsed by the publisher.
